# Importance of cutaneous changes in the diagnosis of neurological diseases

**DOI:** 10.1590/S1679-45082014AI2931

**Published:** 2014

**Authors:** Ana Maria Mateus, Rita Lopes da Silva, Carla Conceição, José Pedro Vieira

**Affiliations:** 1 Pediatrics Service, Hospital do Espírito Santo de Évora, Évora, Portugal.; 2 Neurology Service, Hospital Dona Estefânia, Centro Hospitalar de Lisboa Central, Lisboa, Portugal.; 3 Imaging Service, Hospital Dona Estefânia, Centro Hospitalar de Lisboa Central, Lisboa, Portugal.

**Figure 1:**
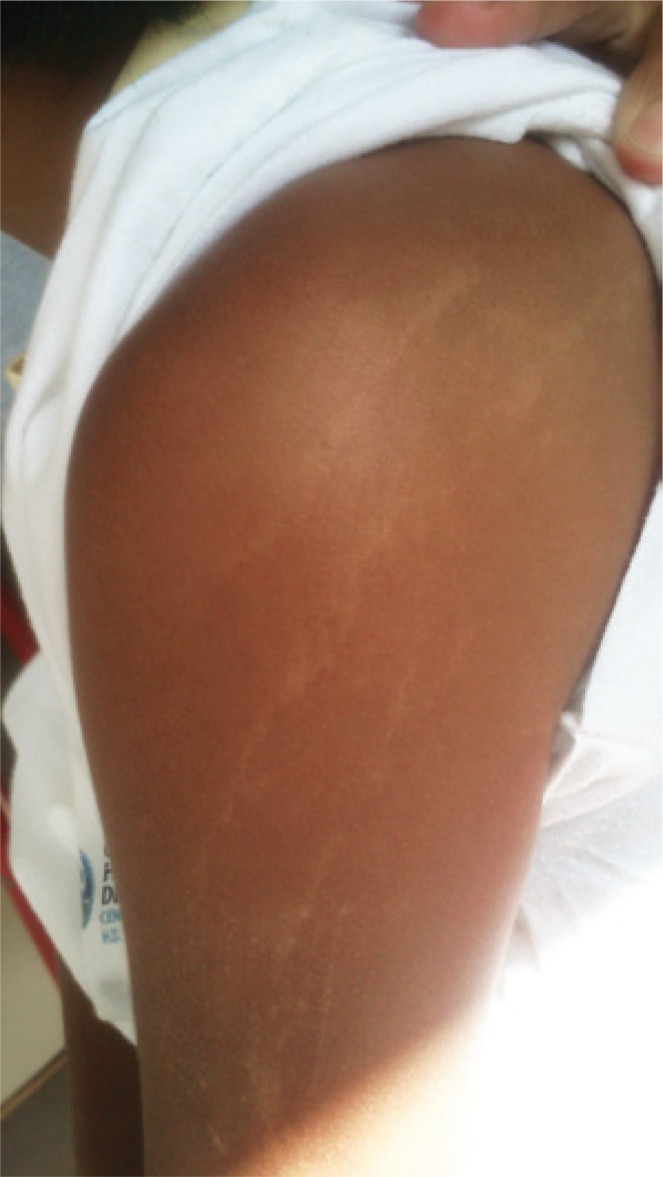
Hypopigmented macules in the upper limb following the lines of Blaschko

**Figure 2:**
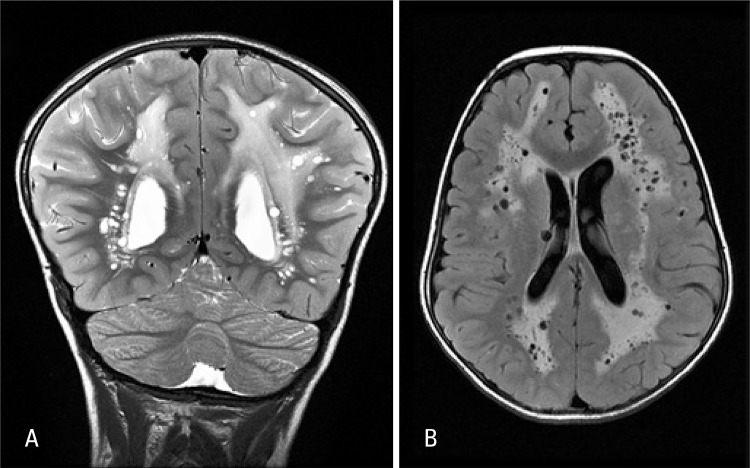
Coronal T2 weighted magnetic resonance (A) and axial fluid attenuated inversion recovery (FLAIR) (B). T2-weighted Hypersignal and FLAIR of white matter on brain hemispheres with periventricular and central predominance in almost symmetric and diffuse manner. Several cysts images spread throughout affected white matter (more linear configured at some areas), which seemed to correspond to dilatation of perivascular spaces

A previous healthy 7-year-old boy presenting occipital headache and constantly wake up at night with 1 week of evolution was admitted to our emergency service. He had linear hypopigmented macules along the lines of Blaschko affecting hemithorax and left upper limbs (Figure 1). The patient also had macrocrania, antimongoloid palpebral fissure, changes in left eye pupil contour and syndactyly. A cranial computed tomography scan showed a hypodensity with accentuation of white matter and small cysts. Cranial resonance revealed an extensive process of leukoencephalopathy and multiple dilatations of perivascular spaces, being such findings rarely described in clinical feature of hypomelanosis of Ito (HI) (Figure 2).

HI is a neurocutaneous syndrome with likelihood to multi-systemic involvement. This syndrome diagnosis presupposes hypopigmented skin lesions in Blaschko’s lines.^(1)^ Most frequent extracutaneous manifestation seen is of neurological origin, especially cognitive impairment and epilepsy.^(2) ^Some cases of minor or null neurological features can show changes in brain resonance.^(3)^ Dilatation of perivascular spaces is a finding of uncertain meaning that is not always associated with neurological disease, mainly in specific areas such as capsulo-lenticular and protuberance. However, multiple perivascular spaces and at unusual locals occur, for example, in mucopolysaccharidosis and in other situations, including in the HI.^(4) ^Other musculoskeletal, craniofacial and ocular manifestations are found in this syndrome, including dysmorphies that are described in our case.^(2,5,6)^

Although a rare disorder, its diagnosis is facilitated by pigmentary mosaicism with morphology and characteristics distribution pattern that reflect different cell lineages.^(2) ^This disease genetic substrate is heterogeneous being described aneuploidies, ring chromosomes, X chromosomal or autosomal inversions and translocations.^(5-8)^

The clinical manifestation variability, early diagnosis and counseling lead to the prognosis.^(5,8) ^Multidisciplinary therapeutic intervention based on clinical findings must be directed to reduce the possible associated morbidity.^(5)^
