# Supplementary material for: Importance of cutaneous changes in the diagnosis of neurological diseases
Source: Einstein (Sao Paulo). 2014 Oct-Dec;12(4):529–30. [Article in Portuguese] doi: 10.1590/S1679-45082014AI2931 (PMC4879927; doi:10.1590/S1679-45082014AI2931)
Supplement: Translation

# Translation: Importância das alterações cutâneas no diagnóstico de doenças neurológicas

**Authors:** Ana Maria Mateus, Rita Lopes da Silva, Carla Conceição, José Pedro Vieira

**Affiliations:** 1 Serviço de Pediatria, Hospital do Espírito Santo de Évora, Évora, Portugal.; 2 Serviço de Neurologia, Hospital Dona Estefânia, Centro Hospitalar de Lisboa Central, Lisboa, Portugal.; 3 Serviço de Imagiologia, Hospital Dona Estefânia, Centro Hospitalar de Lisboa Central, Lisboa, Portugal.

Criança de 7 anos, do gênero masculino, previamente saudável, recorreu ao serviço urgência por cefaleia occipital e despertar noturno com 1 semana de evolução. Apresentava manchas hipopigmentadas lineares segundo as linhas de Blaschko no hemitórax e membro superior esquerdos (Figura 1); macrocrânia, fenda palpebral antimongoloide, alteração do contorno pupilar no olho esquerdo e sindactilia. Sem alterações no exame neurológico. A tomografia computorizada craniencefálica revelou acentuação da hipodensidade da substância branca e diminutas imagens quísticas. A ressonância encefálica evidenciou extenso processo de leucoencefalopatia e dilatações múltiplas de espaços perivasculares, achados raramente descritos no contexto clínico de hipomelanose de Ito (HI) (Figura 2).

**Figura 1:** Manchas hipopigmentadas no membro superior, segundo as linhas de Blaschko

**Figura 2:** Ressonância magnética coronal T2 (A) e *axial fluid attenuated inversion recovery* (FLAIR) (B). Hipersinal T2 e FLAIR da substância branca dos hemisférios cerebrais, com predomínio periventricular e central, de forma quase simétrica e difusa. Incontáveis imagens quísticas dispersas por toda a substância branca lesada, em algumas regiões com configuração mais linear, parecendo corresponder à dilatação de espaços perivasculares

A HI é uma síndrome neurocutânea com potencial de envolvimento multissistémico. O diagnóstico pressupõe lesões cutâneas hipopigmentadas em linhas de Blaschko.^(1)^ As manifestações extracutâneas mais frequentes são do foro neurológico, sobretudo défice cognitivo e epilepsia.^(2)^ Alguns casos com quadro neurológico *minor *ou nulo podem ter alterações na ressonância encefálica.^(3)^ A dilatação de espaços perivasculares é um achado de significado incerto, nem sempre associado a doença neurológica, principalmente quando em regiões específicas, como a capsulolenticular e a protuberância. No entanto, espaços perivasculares múltiplos e fora das localizações habituais ocorrem, por exemplo, nas mucopolissacaridoses e em várias outras situações, incluindo HI.^(4)^ Dessa síndrome fazem também parte manifestações musculoesqueléticas, craniofaciais e oculares nas quais se incluem as dismorfias descritas no caso apresentado.^(2,5,6)^

Embora seja uma afecção rara, seu reconhecimento é facilitado pelo mosaicismo pigmentar, com morfologia e padrão de distribuição característicos, que reflete diferentes linhagens celulares.^(2) ^O substrato genético é heterogêneo, encontrando-se descritas aneuploidias, cromossomos em anel, inversões e translocações de cromossomos X ou de autossomos.^(5-8)^

A variabilidade nas manifestações clínicas e seu diagnóstico e orientação precoces ditam o prognóstico.^(5,8) ^A intervenção terapêutica multidisciplinar, em função dos achados clínicos, deve ser dirigida para minimizar a eventual morbilidade associada.^(5)^
